# Increased carotid intima-media thickness is associated with higher odds of unfavorable outcomes in adults without advanced vascular diseases presenting with non-severe COVID-19 pneumonia: a nested case-control study

**DOI:** 10.3325/cmj.2023.64.344

**Published:** 2023-10

**Authors:** Miljenko Crnjaković, Sabina Deveđija, Gorana Vukorepa, Stela Rutović, Davor Sporiš, Vladimir Trkulja

**Affiliations:** 1 Dubrava University Hospital, Zagreb, Croatia; 2 Department of Pharmacology, Zagreb University School of Medicine, Zagreb, Croatia; * Contributed equally.

## Abstract

**Aim:**

To evaluate the association between carotid intima-media thickness (CIMT) at hospital admission and unfavorable outcomes in adults without advanced vascular diseases presenting with non-severe COVID-19 pneumonia to assess the feasibility of evaluating CIMT as a risk stratification aid in this setting.

**Methods:**

This proof-of-concept nested case-control study enrolled consecutive non-vaccinated adults free of advanced vascular diseases presenting with verified non-severe COVID-19 pneumonia between December 2020 and June 2021. CIMT was measured at admission, and patients were managed in line with the national Ministry of Health guidelines. Those who died or required mechanical ventilation (MV) during the index hospital stay were considered cases and were matched (entropy balancing, exact matching) on a set of covariates to survivors not requiring MV (controls). Frequentist and Bayesian logistic models were fitted to the case status.

**Results:**

The study enrolled 207 patients: 27 (13%) cases and 180 controls. All were retained in the analysis after entropy balancing, while 27 cases were exactly matched to 99 controls. Higher CIMT at the proximal internal carotid artery (both left and right) was consistently associated with higher odds of being a case: all odds ratio point-estimates were ≥1.50 with lower limits of the 99% confidence intervals/credibility intervals ≥1.00 with two-sided probabilities of OR>1.00 greater than 99.5%. The susceptibility of the estimates to unmeasured confounding was low.

**Conclusion:**

This study supports the feasibility of CIMT as a risk stratification aid in adults free of advanced vascular disease presenting with non-severe COVID-19 pneumonia.

COVID-19, an infectious disease caused by the severe acute respiratory syndrome coronavirus-2 (SARS-CoV-2), in up to 20% of symptomatic patients induces viral pneumonia leading to acute respiratory failure (1). In January 2020, the WHO declared the outbreak of COVID-19 to be a public health emergency of international concern (2). High numbers of patients requiring emergency medical services have placed a massive strain on health care systems all over the world. A major problem associated with COVID-19 is incomplete understanding of patterns of disease development. Disease course and outcome cannot be precisely estimated based solely on clinical signs and symptoms (3). Therefore, it would be useful to stratify patients with COVID-19 as having high or low risk for poor outcome at hospital admission. Several predicting scoring systems used commonly in clinical practice, such as Sequential Organ Failure Assessment (SOFA) (4), Modified Early Warning Score (MEWS) (5), and CURB-65 (confusion, uremia, respiratory rate, blood pressure, age ≥65 years) (6), have been investigated in prediction of mortality risk and the risk of requiring mechanical ventilation (MV) during hospital stay in COVID-19 patients (7-10). Although the applicability of these scores in various clinical conditions is indisputable, the COVID-19 pandemic warranted the development of new or additional prognostic tools (10-12) with improved accuracy in identifying, at hospital admission, patients who would subsequently require intensive care unit treatment or suffer poor disease outcomes. Such tools could improve the triage process and patient management in situations of patient overflow and high occupancy of health system capacities.

All of the well-known clinically obvious risk factors for severe forms of COVID-19, ie, intertwined metabolic disorders (eg, dyslipidemia, diabetes, and obesity) and closely accompanying cardiovascular diseases (particularly hypertension) are characterized by endothelial dysfunction (13). Endothelial dysregulation – pre-existing, as well as that induced by the SARS-CoV-2 virus infection (14) – is considered an important cellular driver of the dramatic pulmonary and extrapulmonary events typical for severe COVID-19 (14). In the context of the risk stratification of COVID-19 patients, it might be practical to identify people with endothelial injury among those who still have not experienced cardiovascular incidents resultant from advanced arterial changes. Increased carotid intima-media thickness (CIMT) is an early marker of subclinical atherosclerosis and endothelial injury (15). In this proof-of-concept study, we aimed to evaluate the association between CIMT at admission and a subsequent unfavorable disease course in adults without known advanced vascular diseases presenting with non-severe COVID-19 pneumonia to assess the feasibility of evaluating CIMT as a risk stratification aid in this setting.

## Patients and methods

### Study outline

This nested case-control study enrolled consecutive adults free of advanced vascular diseases and presenting with non-severe COVID-19 pneumonia at the Dubrava University Hospital Respiratory Center between December 2020 and June 2021. The study was approved by the Institutional Review Board of Dubrava University Hospital (2O21/ 2202-02). All patients provided signed informed consent for study inclusion and were managed in line with standard in-house procedures. The only difference was the ultrasound CIMT measurements, which were otherwise not routinely performed. This also included early assessment of the risk of disease deterioration with the MEWS (16) and assessment of pneumonia severity with the CURB-65 score designed for the evaluation of community-acquired pneumonia (17). Patients who died during the index hospital stay or required mechanical ventilation (MV) or extracorporeal membrane oxygenation (ECMO) were considered cases, while the remaining cohort members who were discharged and did not require MV/ECMO were considered controls. We estimated whether higher values of CIMT indicators were associated with the “case” status.

### Inclusion criteria

The study enrolled non-vaccinated adults (≥18 years of age) with confirmed SARS-CoV-2 infection (polymerase chain reaction test) presenting with clinical/radiological signs of pneumonia requiring low-flow oxygen treatment with a nasal cannula at <5 L/min (thus meeting the criteria for hospital admission at our center) who provided informed consent. Patients not requiring oxygen treatment or patients requiring more intense immediate oxygen treatment, patients with clinically manifest or known atherosclerotic cardiovascular diseases (or a history of such a condition), including acute coronary syndrome, myocardial infarction, stable or unstable angina, coronary or other arterial revascularization, stroke, transient ischemic attack, or peripheral artery diseases (including mild-, moderate-, or high-grade carotid artery stenosis), were not enrolled.

### In-house protocol for oxygen therapy

In November 2020, the national Ministry of Health published a second version of the guidelines for the management of patients with COVID-19 (18). These guidelines were followed during the study.

### Antiviral and immunomodulating treatments

Remdesivir was recommended for the treatment of COVID-19 patients with a severe course of disease and illness duration of fewer than 15 days and normal renal function. The use was justified in immunocompromised patients after the 15th day of illness. Patients received 200 mg of remdesivir on day 1, followed by 100 mg once daily for the subsequent 4 to 9 days, depending on their clinical condition. Systemic corticosteroid therapy was recommended in patients with severe and critical COVID-19 after the seventh day of illness. Patients were treated with dexamethasone 10 mg once daily for 10 days. Tocilizumab was considered in patients with clinical worsening and in those at high risk of the cytokine storm syndrome.

Patients were treated with prophylactic enoxaparin (4000 IU subcutaneously) once or twice daily, depending on body weight and renal function (18). Those who developed thrombotic events received therapeutic doses of enoxaparin.

### Intima-media thickness measurement

Carotid ultrasonography was performed within 48 hours of hospital admission with the B- mode carotid ultrasound with Microsoft software (Hitachi Arietta 70, Tokyo, Japan). CIMT was measured at the posterior wall: 1 cm proximally from the most distal part of the right and left main common carotid arteries (CCA); at the carotid bifurcation; and 2 cm distally from the most proximal part of the internal carotid arteries (ACI, *arteria carotis interna*). It corresponds to the space between the two hyperechoic lines on each acquired ultrasound image (19). CIMT was measured by three comparably experienced ultrasonographers (M.C., G.V., and S.R.) depending on their duty schedules.

### Data analysis

We evaluated the association between each of the six CIMT measures – at CCA, at proximal ACI, and at the carotid artery bulb, each on the right and the left side – and the case status at two-sided alpha 0.01 to account for multiplicity arising from six formal evaluations. The full Bonferroni alpha correction would have been too conservative since, with comparisonwise alpha 0.00833, experimentwise alpha is 0.0489. We undertook two procedures to achieve conditional exchangeability between cases and controls regarding covariates other than CIMT indicators: i) entropy balancing (20), a form of distance matching that assigns weights under given enforced restrictions on distance between cases and controls (ie, the distance between moments of covariates), taking into account the estimand (average treatment effect, ATE); ii) exact matching combined with optimal full matching (21,22). The procedure first matches cases and controls (one to many and *vice-versa*) exactly on a set of covariates, and then uses optimal full matching based on Mahalanobis distance to further minimize the distance between the matched units regarding covariates not included in exact matching (21,22). For entropy balancing/matching, the MEWS (0-2 or higher) and CURB-65 (0-1 or higher) scores were dichotomized based on mortality risks associated with their respective values (16,17,23). The procedures were considered successful when standardized differences (d) in covariate values between cases and controls were <0.1. Suboptimally balanced/matched covariates (d ≥0.1) were included in multivariable models. A frequentist and a Bayesian (weighted) logistic model were fitted to case status for each CIMT indicator as a fixed effect in the following ways: i) using raw data (no matching/adjustment), ii) after entropy balancing, and iii) after matching. Frequentist estimation was based on maximum likelihood with robust (entropy balancing) and cluster robust (matching) variance estimators. Bayesian estimation (4 chains, 4000 iterations, 8000 samples of the posterior, highest posterior density intervals) after matching employed hierarchical models to treat subclasses formed by matching as clusters. We defined a moderately informed skeptical normal prior for the association of interest [N(0.0, 0.355) for ln(odds ratio)] consistent with *a priori* hypothesis of no association (assigns 95% probability to an odds ratio [OR] between 0.50 and 2.00), a vaguely informative normal prior for the intercept (0.0, 2.5; scaled), and vaguely informative priors on the terms of a decomposition of the covariance matrices (Gamma shape = 1, scale = 1; LKJ for correlation matrix, regularization = 1; Dirichlet for the simplex vectors, concentration = 1). We used SAS for Windows 9.4 (SAS Inc., Cary, NJ, USA) and the packages *WeightIt* (24), *MatchiIt* (25), and *rstanarm* (26) in the R programming language (27).

### Sample-size calculation

We planned to enroll 200 patients based on the assumptions that around 12% (24 patients) would be cases and that we would be able to match each case to an average of five controls in an exact matching procedure combined with optimal full matching, that is, that there would be 120 controls (successful entropy balancing would retain all cases and controls in the analysis). An odds ratio of 2.00 or higher was considered to indicate a reasonably strong association between CIMT indicators and the case status. Under the assumption of successful matching, and a relative standard deviation of the CIMT measures of 20%-25%, such a sample would provide 87% to 98% power to detect the targeted odds ratio at two-sided alpha = 0.01.

### Susceptibility of unmeasured confounding

To evaluate the susceptibility of the generated estimates to unmeasured confounding, we determined the E-value - the minimum strength of an unmeasured biasing effect (cumulative unmeasured confounding) needed to explain the observed associations between the CIMT indicators and the case status (28), ie, to “push” the estimated OR and its lower limit of the 99% confidence interval/credibility interval (CI/CrI) to 1.00 (“no association”). We also hypothesized the existence of a set of unmeasured covariates with a strong biasing effect (OR = 2.00) with an overall prevalence in the entire cohort of 30% resulting from a large-chance imbalance in the prevalence between cases and controls of 2:1, and we corrected the observed ORs for this hypothetical bias (29). We used the packages *Evalue* (30) and *episensr* (31) in R.

## Results

### Patient characteristics

The cohort comprised 207 patients: 27 (13.0%) cases (23 patients died, 4 required MV but survived) and 180 controls (Table 1). Cases were older than controls, somewhat less frequently men, less frequently obese, and were admitted to hospital sooner after the symptom onset than controls, a finding indicating earlier development of pneumonia (Table 1). The proportions of patients with bilateral pneumonia were comparable (Table 1). The proportions of patients with the MEWS score >2 on-admission and with the CURB-65 score >1 (associated with a higher risk of disease deterioration/mortality) were higher among cases than controls (Table 1). The prevalence of various pre-existing medical conditions was similar, with the exception of hypertension, which was more common in cases (Table 1). All six CIMT measures were higher in cases (Table 1). More cases than controls were subsequently treated with remdesivir, likely due to somewhat more severe disease at presentation. Practically all cases and controls were anticoagulated, and similar (high) proportions were treated with dexamethasone at some point during the disease course (Table 1).

**Table 1:** Patient characteristics: overall, cases, and controls. Data are presented as median (quartiles, minimum-maximum), mean ± standard deviation or count (percent)

	All patients	Cases	Controls	P^†^
N	207	27	180	—
On admission				
Age (years)	67 (57-75; 21-93)	75 (66-84; 47-93)	66 (57-74; 21-93)	<0.001
Men	128 (61.8)	12 (44.4)	116 (64.4)	0.049
BMI<30 kg/m^2^	116 (56.0)	18 (66.7)	98 (54.4)	0.448
BMI≥30 kg/m^2^	101 (44.0)	9 (33.3)	82 (45.5)	—
Symptoms-admission (days)	8 (4-11; 0-35)	4 (2-8; 1-14)	8 (5-12; 0-35)	<0.001
x-ray bilateral pneumonia	33 (15.9)	4 (14.8)	29 (16.1)	0.863
MEWS 0-2 (7.9% risk)	162 (78.3)	17 (63.0)	145 (80.6)	0.050
MEWS 3-6 (12.7%-30% risk)	45 (21.7)	10 (37.0)	35 (19.4)	—
CURB-65 0-1 (0.6%-2.7% risk)	145 (70.1)	14 (51.8)	131 (72.8)	0.030
CURB-65 2-3 (6.8%-14.0% risk)	62 (29.9)	13 (48.2)	49 (27.2)	—
Diabetes	63 (30.4)	9 (33.3)	54 (30.0)	0.727
Hypertension	138 (66.7)	22 (81.5)	116 (64.4)	0.067
Dyslipidemia	28 (13.5)	3 (11.1)	25 (13.9)	0.687
Atrial fibrillation	23 (11.1)	4 (14.8)	19 (10.6)	0.527
COPD or asthma	20 (9.7)	1 (3.7)	19 (10.6)	0.211
Malignancy	21 (10.1)	3 (11.1)	18 (10.0)	0.868
Chronic kidney disease	11 (5.3)	2 (7.4)	9 (5.0)	0.619
Chronic heart failure	4 (1.9)	1 (3.7)	3 (1.7)	0.515
Chronic liver disease^‡^	5 (2.4)	0	5 (2.8)	0.234
CIMT				
right CCA (mm)	0.72 ± 0.16	0.80 ± 0.20	0.71 ± 0.15	0.012
left CCA (mm)	0.73 ± 0.17	0.79 ± 0.22	0.72 ± 0.16	0.034
right carotid bulb (mm)	0.82 ± 0.20	0.91 ± 0.22	0.80 ± 0.19	0.011
left carotid bulb (mm)	0.84 ± 0.20	0.92 ± 0.26	0.83 ± 0.19	0.048
right ACI (mm)	0.65 ± 0.15	0.73 ± 0.18	0.64 ± 0.14	0.011
left ACI (mm)	0.65 ± 0.16	0.74 ± 0.19	0.63 ± 0.15	0.004
Subsequent treatment				
remdesivir	55 (26.6)	11 (40.7)	44 (24.4)	0.085
low molecular weight heparin	195 (94.2)	26 (96.3)	169 (93.9)	0.597
dexamethasone	181 (87.4)	21 (77.8)	160 (88.9)	0.131
intravenous immunoglobulin	6 (2.9)	3 (11.1)	3 (1.7)	0.026
Deaths	23 (11.1)	23 (85.2)	0	—

*Abbreviations: ACI/CCA – internal (*arteria carotis interna*)/common carotid artery; BMI – body mass index; COPD – chronic obstructive pulmonary disease; CIMT – intima-media thickness; MEWS - Modified Early Warning Score.

†Student t, Mann-Whitney or Likelihood ratio test.

‡Chronic hepatitis, cirrhosis, fatty liver disease.

Considering raw covariate data, irrelevant-to-moderate (d = 0.036 to 0.442) and large standardized differences (d = 0.749 for age) were observed between cases and controls (Table 2), and entropy balancing achieved a perfect balance on all covariates (all d <0.05) (Table 2). Matching resulted in 27 cases “paired” to 99 controls with excellent balance (d = 0.000 or <0.1) on all covariates except for a small difference in age (mean 71 vs 69 years, d = 0.239) and the prevalence of the CURB-65 score >1 (37.8% vs 32.7%, d = 0.108) (Table 2).

**Table 2:** Covariates used for entropy balancing and matching (exact combined with optimal full) between cases and controls and intima-media thickness values before and after balancing/matching

	Before balancing/matching			After entropy balancing			After matching		
	cases	controls	d	cases	controls	d	cases	controls	d
N	27	180	—	27	180	—	27	99	—
Covariates									
Age (years)	74 ± 11	65 ± 12	0.749	67 ± 11	66 ± 12	0.042	71 ± 10	69 ± 11	0.239
Men	12 (44.4)	116 (64.4)	-0.410	16.0 (60.7)	111.3 (61.8)	-0.024	13.4 (53.2)	52.6 (53.2)	0.000
BMI<30 kg/m^2^	18 (66.7)	98 (54.4)	0.252	15.0 (55.6)	100.9 (56.0)	-0.009	15.9 (58.7)	58.1 (58.7)	0.000
BMI≥30 kg/m^2^	9 (33.3)	82 (45.5)	-0.252	12.0 (44.4)	79.1 (44.0)	0.009	11.1 (41.3)	40.9 (41.3)	0.000
Diabetes/dyslipidemia	10 (37.0)	65 (36.1)	0.019	1 (37.2)	65.2 (36.2)	0.020	13.1(48.4)	47.9 (48.4)	0.000
Hypertension	22 (81.5)	116 (64.4)	0.391	18.4 (68.1)	120.0 (66.7)	0.033	23.6 (87.4)	86.4 (87.3)	0.000
Other comorbidities†	11 (40.7)	61 (33.9)	0.142	9.1 (33.7)	62.6 (34.8)	-0.022	8.7 (32.3)	33.3 (33.6)	-0.028
Bilateral pneumonia	4 (14.8)	29 (16.1)	-0.036	4.4 (16.3)	28.7 (15.9)	0.010	4.8 (17.7)	15.4 (15.6)	0.059
MEWS score 0-2	17 (63.0)	145 (80.6)	-0.399	21.4 (79.3)	140.9 (78.3)	0.024	21.4 (70.4)	78.6 (79.4)	0.000
MEWS score 3-6	10 (37.0)	35 (19.4)	0.399	5.6 (20.7)	39.1 (21.7)	-0.024	5.6 (20.6)	20.4 (20.6)	0.000
CURB-65 score 0-1	14 (51.8)	131 (72.8)	-0.442	18.7 (69.2)	126.1 (70.0)	-0.017	16.8 (62.2)	66.6 (67.3)	-0.108
CURB-65 score 2-3	13 (48.2)	49 (27.2)	0.442	8.3 (30.8)	53.9 (30.0)	0.017	10.2 (37.8)	32.4 (32.7)	0.108
Intima-media thickness									
right common carotid	0.80 ± 0.20	0.71 ± 0.15	0.469	0.77 ± 0.21	0.72 ± 0.16	0.277	0.81 ± 0.20	0.74 ± 0.17	0.356
left common carotid	0.79 ± 0.22	0.72 ± 0.16	0.390	0.79 ± 0.23	0.72 ± 0.16	0.335	0.84 ± 0.25	0.74 ± 0.16	0.450
right internal carotid bulb	0.91 ± 0.22	0.80 ± 0.19	0.535	0.94 ± 0.21	0.81 ± 0.20	0.637	0.97 ± 0.21	0.85 ± 0.20	0.602
left internal carotid bulb	0.92 ± 0.26	0.83 ± 0.19	0.386	0.97 ± 0.24	0.84 ± 0.19	0.611	0.97 ± 0.23	0.86 ± 0.19	0.509
right internal carotid	0.73 ± 0.18	0.64 ± 0.14	0.555	0.79 ± 0.19	0.64 ± 0.14	0.866	0.79 ± 0.20	0.66 ± 0.14	0.756
left internal carotid	0.74 ± 0.19	0.63 ± 0.15	0.600	0.76 ± 0.20	0.63 ± 0.15	0.705	0.75 ± 0.19	0.63 ± 0.15	0.671

*Abbreviations: BMI - body mass index; MEWS - Modified Early Warning Score.

†Due to the low numbers and similar prevalence in cases and controls (Table 1), atrial fibrillation, chronic obstructive pulmonary disease or asthma, malignancy, chronic kidney or heart failure (none after myocardial infarction) and chronic liver disease were grouped.

### Association between CIMT measures and case status

Higher values of all CIMT measures tended to be associated with higher odds of being a case (all point-estimate ORs >1.00) – based on raw data, after entropy balancing, and after matching (Table 3). However, only for the CIMT measured at the proximal ACI (both on the right and the left side) were the lower limits of the 99%CIs/CrIs consistently ≥1.00 with >99.5% probabilities that the ORs were >1.00 (Table 3). The only exception was the frequentist estimate after entropy balancing pertaining to the left proximal ACI CIMT (OR 1.60, 95%CI 1.04-2.38, 99%CI 0.91-2.71) (Table 3). These estimates appeared unsusceptible to unmeasured confounding – minimum biasing effects needed to “push” the point estimates to 1.00 were well over 2.00, and were well over 1.00 in order to “push” the lower limit of the 99% CI/CrI to 1.00 (except for the left ACI after entropy balancing) (Table 4). Moreover, the OR point-estimates “corrected” for a large hypothetical biasing effect of an unmeasured confounding set with a high imbalance in the prevalence between cases and controls were still well over 1.00 (Table 4).

**Table 3:** Association between indicators of intima-media thickness (CIMT) and the “case” status (death or need for mechanical ventilation or extracorporeal oxygenation) based on raw data (no adjustments), and after entropy covariate balancing or matching (with additional adjustment for age and the CURB-65 score class 0-1 or 2-3). Depicted are odds ratios (ORs) with confidence/credibility intervals (95% and 99%) and two-sided probabilities (frequentist/Bayes) that the ORs were >1.00

	Frequentist estimates			Bayesian estimates		
	OR (95%CI)	99% CI	P (OR>1.0) (%)	OR (95%CrI)	99% CrI	P (OR>1.0) (%)
Raw data						
CCA right	1.34 (1.02-1.77)	0.93-1.94	98.20	1.44 (1.06-1.70)	0.99-1.80	99.36
CCA left	1.27 (0.99-1.61)	0.92-1.74	97.35	1.27 (1.02-1.62)	0.93-1.70	97.66
ACI bulb right	1.31 (1.05-1.64)	0.97-1.76	99.05	1.31 (1.07-1.62)	1.00-1.73	99.64
ACI bulb left	1.24 (0.97-1.57)	0.90-1.69	95.90	1.24 (1.02-1.52)	0.96-1.60	98.22
ACI right	1.45 (1.09-1.93)	1.00-2.11	99.50	1.45 (1.12-1.88)	1.04-2.05	99.71
ACI left	1.45 (1.12-1.89)	1.03-2.05	99.75	1.46 (1.13-1.86)	1.04-1.99	99.92
After balancing						
CCA right	1.20 (0.81-1.78)	0.71-2.02	81.75	1.34 (0.96-2.34)	0.85-2.83	96.12
CCA left	1.22 (0.89-1.67)	0.81-1.85	89.70	1.42 (0.95-2.16)	0.89-2.77	96.35
ACI bulb right	1.37 (1.01-1.85)	0.92-2.03	97.95	1.55 (1.12-2.27)	0.99-2.59	99.62
ACI bulb left	1.40 (1.01-1.92)	0.91-2.13	97.90	1.46 (1.05-2.05)	0.96-2.39	99.10
ACI right	1.77 (1.17-2.69)	1.02-3.07	99.65	2.03 (1.31-3.22)	1.20-4.01	99.98
ACI left	1.60 (1.04-2.38)	0.91-2.71	98.30	1.78 (1.18-2.80)	1.07-3.39	99.85
After matching						
CCA right	1.20 (0.821.75)	0.73-1.98	83.35	1.21 (0.93-1.58)	0.84-1.70	92.00
CCA left	1.27 (0.99-1.63)	0.91-1.77	97.10	1.29 (1.01-1.63)	0.94-1.80	98.49
ACI bulb right	1.32 (0.98-1.78)	0.89-1.96	96.70	1.35 (1.09-1.70)	1.02-1.82	99.78
ACI bulb left	1.29 (0.96-1.73)	0.88-1.91	95.70	1.30 (1.04-1.65)	0.97-1.77	98.88%
ACI right	1.66 (1.23-2.24)	1.11-2.46	99.95	1.71 (1.26-2.27)	1.17-2.53	99.98
ACI left	1.50 (1.17-1.93)	1.08-2.09	99.90	1.53 (1.15-2.01)	1.05-2.18	99.89

*Abbreviations: CCA – common carotid artery; ACI – internal carotid artery (*arteria carotis interna*); OR - odds ratio; CI - confidence interval; CrI - credibility interval.

**Table 4:** Sensitivity to unmeasured confounding of the estimates of the association between intima-media thickness at the proximal internal carotid artery (ACI) (right and left) and the case status (death or need for mechanical ventilation or extracorporeal oxygenation). Shown are observed odds ratios (OR) with lower limit (LL) of the 99% confidence interval/credibility interval (CI/CrI); E-values, ie, minimum strength (on a relative risk scale) of a (cumulative) confounding effect needed to “push” the OR point-estimate and LLs of the 99% CI/CrI to 1.00 and “bias-corrected” OR – corrected for a hypothetical strong biasing effect (OR = 2.00) of an unmeasured confounder considerably more prevalent in cases (52%) than in controls (26%)

	Observed OR (LL 99%)	E-value for OR (LL) to 1.00	Bias-corrected OR
After entropy balancing			
ACI right (frequentist)	1.77 (1.02)	2.93 (1.16)	1.47
ACI right (Bayes)	2.03 (1.20)	3.47 (1.69)	1.68
ACI left (frequentist)	1.60 (0.91)	2.58 (1.00)	1.33
ACI left (Bayes)	1.78 (1.07)	2.96 (1.34)	1.48
After matching			
ACI right (frequentist)	1.66 (1.11)	2.71 (1.46)	1.38
ACI right (Bayes)	1.71 (1.17)	2.81 (1.62)	1.42
ACI left (frequentist)	1.50 (1.08)	2.37 (1.37)	1.24
ACI left (Bayes)	1.53 (1.05)	2.43 (1.28)	1.27

*Abbreviations: ACI – internal carotid artery (*arteria carotis interna*).

## Discussion

In the present proof-of-concept study, CIMT measurements were shown to be candidates for a more comprehensive evaluation of their utility in the risk stratification of patients with milder forms of COVID-19 pneumonia but free of advanced clinically obvious vascular diseases. To the best of our knowledge, this is the first study to address this issue. The rationale behind it comprises several elements: i) COVID-19 is characterized by a highly unpredictable course and outcomes (32), which complicates treatment decisions in individual patients and the organizational aspects of the health care system; ii) the usefulness of known risk scoring systems commonly used in severely ill patients, eg, SOFA, MEWS, and CURB-65 (9,10,33-35), is limited in this respect and further improvements have been warranted (35); iii) considering the nature of COVID-19, the predictive value of a certain indicator might differ in patients at different stages of the disease. We focused on one specific subset of COVID-19 patients, ie, those presenting at an early or milder stage of pneumonia who are not explicitly burdened with advanced vascular diseases; iv) CIMT measurement is a simple, well-established, and readily available method that could be performed at the bedside. It is a well-known indicator of endothelial dysfunction (36), the main driver of rapidly progressing severe or fatal COVID-19 forms (37-41) and a common denominator in several conditions that are “classical” risk factors for untoward outcomes (eg, inter-related metabolic and cardiovascular conditions) (41-44); v) a proper evaluation of a potential “indicator of future events” is an extensive and demanding undertaking; one should first assess whether it would be feasible at all. The main present finding is a clear-cut independent association between a higher CIMT at the posterior wall of the proximal part of the internal carotid arteries and higher odds of death or need for MV during the index hospital stay. These observations should be considered in light of the study limitations. Nested case-control studies allow one to adjust for potential confounders as assessed at the inception of the cohort, but potential confounding between the start of observation and occurrence of the outcome cannot be adequately accounted for. For example, treatments introduced during the index hospital admission based on subsequent clinical status might have influenced the outcome. However, an attempt to adjust for this post-baseline confounding is likely to result in collider bias since treatments were likely affected by both the “exposure” (CIMT at admission) and the outcome (disease severity at a certain point in time) (45). In this respect, it should be noted that all patients were managed in line with the recommendations of the national Ministry of Health (18), which remained unchanged during the study period (around six months). Next, we did not account for a number of other indicators of endothelial dysfunction (46,47). However, we did not aim to study the role of endothelial dysfunction – its role in COVID-19 has been extensively elaborated (38-41). The fact that CIMT is one of the known indicators of this process just provided a rationale for its evaluation for the present purpose. Finally, the present analysis was conducted on a limited single-center sample. On the other hand, we accounted for a number of patients’ characteristics known to be associated with unfavorable COVID-19 course, including the CURB-65 and MEWS scores shown as predictive of poor COVID-19 outcomes (10,34,48). Moreover, the present estimates appeared reasonably “resistant” to unmeasured confounding - very strong confounding effects would have been needed to explain the observed associations, while estimates corrected for substantial hypothetical biasing effects still indicated a rather strong association between higher IMT at proximal ACI and higher odds for death or need for MV.

Overall, the present findings document an independent association between higher IMT (dorsal wall) at the proximal part of the internal carotid arteries and higher odds of death or need for MV in adult COVID-19 patients presenting with non-severe pneumonia who are not burdened with clinically explicit advanced cardio- or cerebrovascular diseases. Consequently, it appears feasible to evaluate CIMT measurements as aids in risk stratification in these patients, and, potentially, in other COVID-19 patient subsets. One practical obstacle in this effort is easily envisaged: despite the fact that CIMT has been long investigated in cardiovascular pathology settings, the cut-off value of “abnormal” CIMT is debatable because the reference ranges are age- and sex-dependent, with significantly higher values in men and an age-related increase in all carotid segments (49-51). Professional guidelines have suggested CIMT>0.9 as a marker of asymptomatic organ damage or values ≥75th percentile for the respective age and sex as indicative of an increased cardiovascular risk (52). These values might be considered as starting points in the evaluation of CIMT in risk stratification in COVID-19 patients.

In conclusion, the present study supports the feasibility of evaluating CIMT as a risk stratification aid in adult COVID-19 patients presenting with milder COVID-19-related pneumonia.

## References

[1] Zhu J Ji P Pang J Zhong Z Li H He C Clinical characteristics of 3062 COVID-19 patients: A meta-analysis. J Med Virol 2020 92 1902 14 10.1002/jmv.25884 32293716 PMC7262119

[2] Sohrabi C Alsafi Z O’Neill N Khan M Kerwan A Al-Jabir A World Health Organization declares global emergency: A review of the 2019 novel coronavirus (COVID-19). Int J Surg 2020 76 71 6 10.1016/j.ijsu.2020.02.034 32112977 PMC7105032

[3] Wynants L Van Calster B Collins GS Riley RD Heinze G Schuit E Prediction models for diagnosis and prognosis of covid-19: systematic review and critical appraisal. BMJ 2020 369 m1328 10.1136/bmj.m1328 32265220 PMC7222643

[4] Vincent JL Moreno R Takala J Willatts S De Mendonça A Bruining H The SOFA (Sepsis-related Organ Failure Assessment) score to describe organ dysfunction/failure. On behalf of the Working Group on Sepsis-Related Problems of the European Society of Intensive Care Medicine. Intensive Care Med 1996 22 707 10 10.1007/BF01709751 8844239

[5] Subbe CP Kruger M Rutherford P Gemmel L Validation of a modified Early Warning Score in medical admissions. QJM 2001 94 521 6 10.1093/qjmed/94.10.521 11588210

[6] Lim WS van der Eerden MM Laing R Boersma WG Karalus N Town GI Defining community acquired pneumonia severity on presentation to hospital: an international derivation and validation study. Thorax 2003 58 377 82 10.1136/thorax.58.5.377 12728155 PMC1746657

[7] Wang L Lv Q Zhang X Jiang B Liu E Xiao C The utility of MEWS for predicting the mortality in the elderly adults with COVID-19: a retrospective cohort study with comparison to other predictive clinical scores. PeerJ 2020 8 e10018 10.7717/peerj.10018 33062437 PMC7528814

[8] Zhou F Yu T Du R Fan G Liu Y Liu Z Clinical course and risk factors for mortality of adult inpatients with COVID-19 in Wuhan, China: a retrospective cohort study. Lancet 2020 395 1054 62 10.1016/S0140-6736(20)30566-3 32171076 PMC7270627

[9] Liu S Yao N Qiu Y He C Predictive performance of SOFA and qSOFA for in-hospital mortality in severe novel coronavirus disease. Am J Emerg Med 2020 38 2074 80 10.1016/j.ajem.2020.07.019 33142178 PMC7354270

[10] Lucijanić M Piskač Živković N Režić T Durlen I Stojić J Jurin I The performance of the WHO COVID-19 severity classification, COVID-GRAM, VACO Index, 4C Mortality and CURB65 prognostic scores in hospitalized COVID-19 patients: data on 4014 patients from a tertiary center registry. Croat Med J 2023 64 13 20 10.3325/cmj.2023.64.13 36864814 PMC10028560

[11] Roth GA Emmons-Bell S Alger HM Bradley SM Das SR de Lemos JA Trends in Patient Characteristics and COVID-19 In-Hospital Mortality in the United States During the COVID-19 Pandemic. JAMA Netw Open 2021 4 e218828 10.1001/jamanetworkopen.2021.8828 33938933 PMC8094014

[12] Peckham H de Gruijter NM Raine C Radziszewska A Ciurtin C Wedderburn LR Male sex identified by global COVID-19 meta-analysis as a risk factor for death and ITU admission. Nat Commun 2020 11 6317 10.1038/s41467-020-19741-6 33298944 PMC7726563

[13] Sanchis-Gomar F Lavie CJ Mehra MR Henry BM Lippi G Obesity and Outcomes in COVID-19: When an Epidemic and Pandemic Collide. Mayo Clin Proc 2020 95 1445 53 10.1016/j.mayocp.2020.05.006 32622449 PMC7236707

[14] Bernard I Limoneta D Mahal LK HObman TC. Endothelium infection and dysregulation by SARS-CoV-2: evidence and caveats in COVID-19. Viruses 2020 13 29 10.3390/v13010029 33375371 PMC7823949

[15] Sibal L Agarwal SC Home PD Carotid intima-media thickness as a surrogate marker of cardiovascular disease in diabetes. Diabetes Metab Syndr Obes 2011 4 23 34 10.2147/DMSO.S8540 21448319 PMC3064409

[16] Subbe CP Kruger M Rutherford P Gemmel L Validation of a modified Early Warning Score in medical admissions. QJM 2002 94 521 6 10.1093/qjmed/94.10.521 11588210

[17] Lim WS van der Eerden MM Laing R Boersma WG Karalus N Town GI Defining community acquired pneumonia severity on presentation to hospital: an international derivation and validation study. Thorax 2003 58 377 82 10.1136/thorax.58.5.377 12728155 PMC1746657

[18] Ministry of Health of the Republic of Croatia. COVID – 19 Treatment Guidelines. Version 2. November 19, 2020. Available from: https://zdravlje.gov.hr/UserDocsImages/2020%20CORONAVIRUS/Smjernice%20za%20lije%C4%8Denje%20oboljelih%20od%20koronavirusne%20bolesti%202019%20(COVID-19),%20verzija%202%20od%2019.%20studenoga%202020.pdf*.* Accessed November 30, 2021.

[19] Nezu T Hosomi N Aoki S Matsumoto M Carotid intima-media thickness for atherosclerosis. J Atheroscler Thromb 2016 23 18 31 10.5551/jat.31989 26460381

[20] Hainmueller J Entropy balancing for causal effects: a multivariate reweighting method to. produce balanced samples in observational studies. Polit Anal 2012 20 25 46 10.1093/pan/mpr025

[21] King G Nielsen R Why propensity scores should not be used for matching. Polit Anal 2019 27 435 54 10.1017/pan.2019.11

[22] Hansen BB Olsen Klopfer S Optimal full matching and related designs via network flows. J Comput Graph Stat 2006 15 609 27 10.1198/106186006X137047

[23] Aujesky D Auble TE Yealy DM Stone RA Obrosky DS Meehan TP Prospective comparison of three validated prediction rules for prognosis in community-acquired pneumonia. Am J Med 2005 118 384 92 10.1016/j.amjmed.2005.01.006 15808136

[24] Greifer N. WeightIt: Weighting for covariate balance in observational studies. 2022. Available from: https://ngreifer.github.io/WeightIt/, https://github.com/ngreifer/WeightIt. Accessed: September 18, 2023.

[25] Ho DE Imai K King G Stuart EA MatchIt: nonparametric preprocessing for parametric causal inference. J Stat Softw 2011 42 i08 10.18637/jss.v042.i08

[26] Goodrich B, Gabry J, Ali I, Brilleman S. rstanarm: Bayesian applied regression modeling via Stan. R package version 2.21.3, 2022. Available from: https://mc-stan.org/rstanarm/. Accessed: September 18, 2023.

[27] R Core Team. R: A language and environment for statistical computing. R Foundation for Statistical Computing, Vienna, Austria, 2020.

[28] VanderWeele TJ Ding P Sensitivity analysis in observational research: introducing the E-value. Ann Intern Med 2017 167 268 74 10.7326/M16-2607 28693043

[29] Schneeweiss S Sensitivity analysis and external adjustment for unmeasured confounders in epidemiologic database studies of therapeutics. Pharmacoepidemiol Drug Saf 2006 15 291 303 10.1002/pds.1200 16447304

[30] Mathur MB Ding P VanderWeele TJ Website and R package for computing E-values. Epidemiology 2018 29 e45 7 10.1097/EDE.0000000000000864 29912013 PMC6066405

[31] Heine D. The episensr package: basic sensitivity analysis of epidemiological results. R package version 1.1.0. Available from: https://dhaine.github.io/episensr/. September 18, 2023.

[32] Attaway AH Scheraga RG Bhimraj A Biehl M Hatipoğlu U Severe covid-19 pneumonia: pathogenesis and clinical management. BMJ 2021 372 10.1136/bmj.n436 33692022

[33] Raschke RA Agarwal S Rangan P Heise CW Curry SC Discriminant accuracy of the sofa score for determining the probable mortality of patients with COVID-19 pneumonia requiring mechanical ventilation. JAMA 2021 325 1469 70 10.1001/jama.2021.1545 33595630 PMC7890534

[34] Guo J Zhou B Zhu M Yuan Y Wang Q Zhou H CURB-65 may serve as a useful prognostic marker in COVID-19 patients within Wuhan, China: a retrospective cohort study. Epidemiol Infect 2020 148 e241 10.1017/S0950268820002368 32998791 PMC7573457

[35] Coughlan C Rahman S Honeyford K Costelloe CE Developing useful early warning and prognostic scores for COVID-19. Postgrad Med J 2021 97 477 80 10.1136/postgradmedj-2021-140086 37066681 PMC10016951

[36] Halcox JP Donald AE Ellins E Witte DR Shipley MJ Brunner EJ Endothelial function predicts progression of carotid intima-media thickness. Circulation 2009 119 1005 12 10.1161/CIRCULATIONAHA.108.765701 19204308

[37] Ackermann M Verleden SE Kuehnel M Haverich A Welte T Laenger F Pulmonary vascular endothelialitis, thrombosis, and angiogenesis in Covid-19. N Engl J Med 2020 383 120 8 10.1056/NEJMoa2015432 32437596 PMC7412750

[38] Nägele MP Haubner B Tanner FC Ruschitzka F Flammer AJ Endothelial dysfunction in COVID-19: Current findings and therapeutic implications. Atherosclerosis 2020 314 58 62 10.1016/j.atherosclerosis.2020.10.014 33161318 PMC7554490

[39] Varga Z Flammer AJ Steiger P Haberecker M Andermatt R Zinkernagel AS Endothelial cell infection and endotheliitis in COVID-19. Lancet 2020 395 1417 8 10.1016/S0140-6736(20)30937-5 32325026 PMC7172722

[40] Goshua G Pine AB Meizlish ML Chang CH Zhang H Bahel P Endotheliopathy in COVID-19-associated coagulopathy: evidence from a single-centre, cross-sectional study Lancet Haematol 2020 7 e575 82 10.1016/S2352-3026(20)30216-7 32619411 PMC7326446

[41] Poor HD Ventetuolo CE Tolbert T Chun G Serrao G Zeidman A COVID-19 critical illness pathophysiology driven by diffuse pulmonary thrombi and pulmonary endothelial dysfunction responsive to thrombolysis. Clin Transl Med 2020 10 e44 10.1002/ctm2.44 32508062 PMC7288983

[42] CDC COVID-Response Team preliminary estimates of the prevalence of selected underlying health conditions among patients with coronavirus disease 2019 - United States, February 12-March 28, 2020 MMWR Morb Mortal Wkly Rep 2020 69 382 6 10.15585/mmwr.mm6913e2 32240123 PMC7119513

[43] Zhou F Yu T Du R Fan G Liu Y Liu Z Clinical course and risk factors for mortality of adult inpatients with COVID-19 in Wuhan, China: a retrospective cohort study Lancet 2020 395 1054 62 10.1016/S0140-6736(20)30566-3 32171076 PMC7270627

[44] Guo T Fan Y Chen M Wu X Zhang L He T Cardiovascular implications of fatal outcomes of patients with coronavirus disease 2019 (COVID-19) JAMA Cardiol 2020 5 811 8 10.1001/jamacardio.2020.1017 32219356 PMC7101506

[45] Griffith GJ Morris TT Tudball MJ Herbert A Mancano G Pike L Collider bias undermines our understanding of COVID-19 disease risk and severity. Nat Commun 2020 11 5749 10.1038/s41467-020-19478-2 33184277 PMC7665028

[46] Flammer AJ Anderson T Celermajer DS Creager MA Deanfield J Ganz P The assessment of endothelial function: from research into clinical practice. Circulation 2012 126 753 67 10.1161/CIRCULATIONAHA.112.093245 22869857 PMC3427943

[47] Järvisalo MJ Juonala M Raitakari OT Assessment of inflammatory markers and endothelial function. Curr Opin Clin Nutr Metab Care 2006 9 547 52 10.1097/01.mco.0000241663.00267.ae 16912549

[48] Hu H Yao N Qiu Y Predictive value of 5 early warning scores for critical COVID-19 patients. Disaster Med Public Health Prep 2020 16 232 9 10.1017/dmp.2020.324 32900406 PMC7596567

[49] Tosetto A Prati P Baracchini C Manara R Rodeghiero F Age-adjusted reference limits for carotid intima-media thickness as better indicator of vascular risk: population-based estimates from the VITA project. J Thromb Haemost 2005 3 1224 30 10.1111/j.1538-7836.2005.01440.x 15946212

[50] Lorenz MW von Kegler S Steinmetz H Markus HS Sitzer M Carotid intima-media thickening indicates a higher vascular risk across a wide age range: prospective data from the Carotid Atherosclerosis Progression Study (CAPS). Stroke 2006 37 87 92 10.1161/01.STR.0000196964.24024.ea 16339465

[51] Touboul PJ Labreuche J Vicaut E Belliard JP Cohen S Kownator S PARC Study Investigators Country-based reference values and impact of cardiovascular risk factors on carotid intima-media thickness in a French population: the ‘Paroi Artérielle et Risque Cardio-Vasculaire’ (PARC) Study. Cerebrovasc Dis 2009 27 361 7 10.1159/000202013 19218802

[52] Stein JH Korcarz CE Hurst RT Lonn E Kendall CB Mohler ER Use of carotid ultrasound to identify subclinical vascular disease and evaluate cardiovascular disease risk: a consensus statement from the American Society of Echocardiography Carotid Intima-Media Thickness Task Force. Endorsed by the Society for Vascular Medicine. J Am Soc Echocardiogr 2008 21 93 111 10.1016/j.echo.2007.11.011 18261694

